# Structural characteristics and antiviral activity of multiple peptides derived from MDV glycoproteins B and H

**DOI:** 10.1186/1743-422X-8-190

**Published:** 2011-04-25

**Authors:** Xiaojia Wang, Xiaojing Chi, Ming Wang

**Affiliations:** 1 Key Laboratory of Zoonosis of Ministry of Agriculture, College of Veterinary Medicine, China Agricultural University, No. 2, Yuan Ming Yuan West Road, Haidian District, Beijing 100193, PR China

**Keywords:** Marek's disease virus, glycoprotein, plaque formation, chorioallantoic membrane, structural characteristics, antiviral inhibitor, viral entry mechanism

## Abstract

**Background:**

Marek's disease virus (MDV), which is widely considered to be a natural model of virus-induced lymphoma, has the potential to cause tremendous losses in the poultry industry. To investigate the structural basis of MDV membrane fusion and to identify new viral targets for inhibition, we examined the domains of the MDV glycoproteins gH and gB.

**Results:**

Four peptides derived from the MDV glycoprotein gH (gHH1, gHH2, gHH3, and gHH5) and one peptide derived from gB (gBH1) could efficiently inhibit plaque formation in primary chicken embryo fibroblast cells (CEFs) with 50% inhibitory concentrations (IC_50_) of below 12 μM. These peptides were also significantly able to reduce lesion formation on chorioallantoic membranes (CAMs) of infected chicken embryos at a concentration of 0.5 mM in 60 μl of solution. The HR2 peptide from Newcastle disease virus (NDVHR2) exerted effects on MDV specifically at the stage of virus entry (i.e., in a cell pre-treatment assay and an embryo co-treatment assay), suggesting cross-inhibitory effects of NDV HR2 on MDV infection. None of the peptides exhibited cytotoxic effects at the concentrations tested. Structural characteristics of the five peptides were examined further.

**Conclusions:**

The five MDV-derived peptides demonstrated potent antiviral activity, not only in plaque formation assays in vitro, but also in lesion formation assays in vivo. The present study examining the antiviral activity of these MDV peptides, which are useful as small-molecule antiviral inhibitors, provides information about the MDV entry mechanism.

## Background

The entry of enveloped viruses into host cells occurs via fusion of the viral envelope with the cellular membrane. This membrane fusion is mediated by several glycoproteins in the viral envelope that overcome strong repulsive hydration forces as well as steric and electrostatic barriers. Several of the functional motifs present in different viral fusion glycoproteins are drug development targets [1].

Herpesviruses are structurally complex enveloped viruses that have at least twelve glycoproteins on their surfaces. Unlike orthomyxoviruses, paramyxoviruses, filoviruses, and retroviruses, which all use a single fusion glycoprotein for membrane fusion, herpesviruses use a conserved core fusion machinery consisting of the glycoprotein gB and a gH-gL heterodimer [2]. gB is a class III viral fusion protein, also called a fusogen, that is presumably directly involved in bringing the viral and cellular membranes together but cannot function on its own [3,4]. The crystal structure of the gH ectodomain bound to gL shows an unusually tight complex with a unique architecture; and the formation of a gB-gH-gL complex is critical for membrane fusion [5]. The fusion machinery of herpesviruses is more complex than that of most enveloped viruses and is somewhat reminiscent of the fusion machinery involved in cellular fusion processes [6-9]. In some herpesviruses, both gH and gB possess heptad repeat (HR) regions, and the peptides from HR regions have been shown to inhibit fusion [10-12]. Furthermore, it has been shown that the α-helix rich and hydrophobic regions of viral fusion proteins may be required for efficient induction of fusion [13-16].

Marek's disease virus (MDV) has long been of interest as a model organism, particularly with respect to the pathogenesis and immune control of virus-induced lymphoma in an easily accessible small animal system. MDV was long thought to be related to Epstein-Barr virus (EBV), a member of the *Gammaherpesvirinae *family, owing to its biological properties, particularly its slow growth in culture and its ability to induce T-cell lymphoma. Electron microscopy studies of the MDV genome provided the first evidence that this double-stranded DNA virus possesses repeat structures that are characteristic of the *Alphaherpesvirinae*, which was later confirmed by detailed restriction mapping and sequencing of individual genes and then entire genomes. It is now known that MDV is genetically closely related to human herpesvirus 1 (herpes simplex virus type 1, HSV-1) and human herpesvirus 3 (varicella-zoster virus, VZV) [17]. Recent advances in MDV genetics and the sequencing of the chicken genome, aided by functional genomics have increased our understanding of lytic MDV replication and the factors and mechanisms leading to latency and tumour formation [17,18]. MDV is found in all areas of the world and particularly virulent forms of this virus frequently cause acute explosive outbreaks, despite the availability of vaccines. The non-oncogenic MDV strains used as a vaccine prevent tumour growth but do not prevent the replication of either vaccine or virulent strains, and infectious virus particles survive at room temperature for several months [19]. To understand the molecular mechanisms of MDV entry into host cells and to potentially identify inhibitory agents, we sought to determine the functional roles of specific regions of gH and gB proteins involved in the membrane fusion process [20,21].

## Results

### MDV gH and gB have serial HR regions showing potential antiviral activity

**Figure 1:**
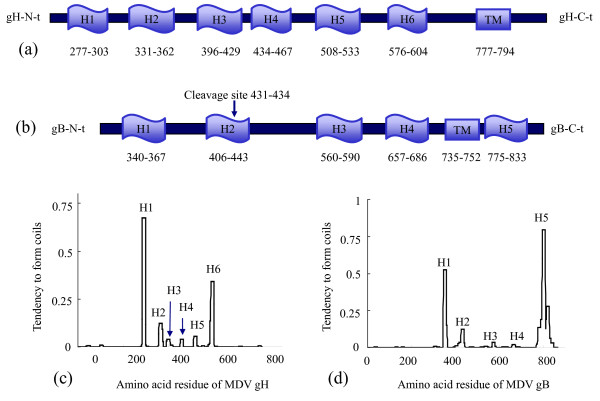
**Prediction of functional domains of MDV gH and gB**. **(a) **Linear representation of the MDV gH glycoprotein including potential HR regions and transmembrane region (TM). **(b) **Linear representation of the MDV gB glycoprotein including potential HR regions and TM region. gBH2 domain contains cleavage site located at aa residues 431 to 434. **(c) **Six HR domains of MDV gH as predicted by the ExPASy-Coils program. **(d) **Five HR domains of MDV gB as predicted by the ExPASy-Coils program.

**Figure 2:**
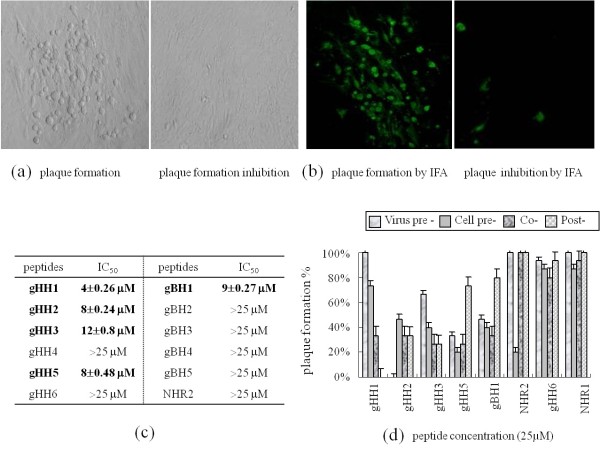
**Effect of peptides on plaque formation**. **(a) **Photos show plaque formation at 10 × 10 magnification (infected by 100 pfu of MDV without peptides, i.e., 100% plaque formation) and 0% plaque formation (infected by 100 pfu MDV with 50 μM gHH2 in cell-pre treatment assay, i.e., 100% plaque formation inhibition). **(b) **The plaque formation using immunofluorescent (IFA) staining with 1:100 diluted antibody anti-pp24, and plaque formation inhibition are shown from left to right, respectively. **(c) **Five peptides (gHH1, gHH2, gHH3, gHH5, and gBH1) with IC_50 _values below 12 μM were selected for further study. In addition, NHR2 (i.e. NDV HR2) and gHH6 were selected as control proteins in the paper. Note: The IC_50 _are the means ± standard deviations determined from three independent experiments. **(d) **CEF cells were exposed to peptides at concentration of 25 μM either prior to infection (cell pre-treatment, cell pre-), during entry (co-treatment, co-) or after virus penetration (post-treatment, post-), or alternatively, the virus was pre-incubated with peptides for 1 h at 37°C before addition to the cells (virus pre-treatment, virus pre-). Experiments were performed in triplicate, and the plaque formation percentages were calculated. Peptides gHH1, gHH2, gHH3, gHH5, gBH1, NHR2, gHH6, and NHR1 (i.e. NDV HR1) are shown from left to right, respectively.

In this study, we searched for HR regions in MDV gH and gB proteins using a series of biological software packages. Six potential HR regions in gH and five potential HR regions in gB were identified (see Figure 1). To determine whether these peptides could affect virus infectivity, primary CEFs were incubated with peptides at increasing concentrations in the presence of 100 pfu MDV (i.e., the co-treatment assay). The cells were then incubated for 5 days at 37°C in DMEM supplemented with 2% FCS, and plaques were counted. Plaque formation is shown in Figure 2a. We also used an immunofluorescence (IFA) assay with anti-pp24 antibody to verify plaque formation [22] (see Figure 2b). Uninfected cells resembled cells in which plaque formation was inhibited, and they are not shown here. These experiments demonstrate that peptides gHH4, gHH6, and gBH2-5 could inhibit plaque formation at an IC_50 _of more than 25 μM. Five peptides (gHH1, gHH2, gHH3, gHH5, and gBH1) with IC_50 _values below 12 μM were selected for further studies. The IC_50 _values of gHH1, gHH2, gHH3, gHH5, and gBH1 were approximately 4, 8, 12, 8 and 9 μM, respectively. These results are shown in Figure 2c. In addition, gHH6 was considered for further analysis in current study because its hydrophilic character is similar to the most potent inhibitor gHH1, and they were two HR domains with the highest predicted tendency to form coiled-coil structures (see Figure 1c).

**Figure 3:**
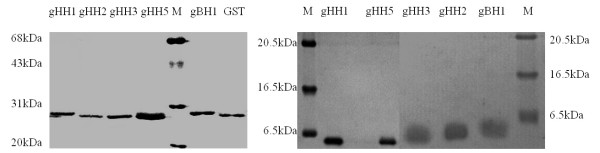
**SDS-PAGE analyses of GST-fusion proteins and cleaved peptides by protease 3C**. **(left) **From left to right, purified GST-gHH1 protein, purified GST-gHH2 protein, purified GST-gHH3 protein, purified GST-gHH5 protein, protein molecular mass markers (20, 31, 43, 68, 97 kDa), purified GST-gBH1 protein, and GST protein. **(right) **From left to right, protein molecular marker (7, 17, 25 kDa), purified gHH1 peptide after GST-3C cleavage, gHH5 after GST-3C cleavage, gHH3 after GST-3C cleavage, gHH2 after GST-3C cleavage, gBH1 after GST-3C cleavage, markers (7, 17, 25 kDa).

All of the GST fusion proteins used in this study were expressed as soluble proteins. The cleaved proteins were highly soluble in PBS at concentrations of about 1 mg/ml. SDS-PAGE gels of the five GST fusion proteins and corresponding cleaved proteins are shown in Figure 3.

### gHH1, gHH2, gHH3, gHH5, and gBH1 have potent antiviral activities at different steps of the viral entry process

A plaque formation experiment was conducted to identify which steps in the entry process were inhibited by gB- and gH-derived peptides at a concentration of 25 μM, the concentration which induced significant inhibition, and to compare the effect of different four methods without a strong bias. Four different assays were conducted: cells were exposed to peptides at different concentrations prior to infection (cell pre-treatment), during entry (co-treatment), after viral entry (post-treatment), or when the virus was pre-incubated with the peptide for 1 hour at 37°C before attaching to the cells (virus pre-treatment). After all treatments, the cells were incubated for 5 days at 37°C in DMEM supplemented with FCS and plaque numbers were then determined. All five peptides showed potent antiviral activity in the co-treatment experiment and inhibited infection to a minor extent in other assays. These results demonstrate that at 25 μM, gHH1 was the most effective peptide in the post-treatment assay with 100% inhibition of plaque formation. Plaque formation was completely inhibited by the gHH2 peptide at 25 μM in a virus pre-treatment assay. In addition, all five peptides tested inhibited plaque formation 60-80% in the co-treatment assay at a concentration of 25 μM. Furthermore, plaque formation was completely inhibited by gHH1 at 50 μM in the co-treatment assay and plaque formations of gHH2, gHH3, gHH5, and gBH1 were nearly 20%, 20%, 13%, and 12% at 50 μM. These results indicate that gHH1 and gHH2 should be the most effective peptides from this study for small-molecule antiviral drug design to inhibit MDV entry. Finally, the HR2 region from the fusion glycoprotein (gF) of Newcastle disease virus (NDV) (NDVHR2) at 25 μM demonstrated antiviral activity with 20% plaque formation, more effective than MDV-derived peptides, when used prior to MDV entry into cells (i.e., in the cell pre-treatment assay)." NDVHR1 and gHH6 (the negative controls) did not show significant antiviral activity, demonstrating the specificity of the antiviral effect of the MDV-derived peptides used in this study. These results are shown in Figure 2d.

### None of the peptides exhibit cytotoxic effects

To confirm that these peptides did not exert toxic effects on CEF cells, cell monolayers were exposed to a range of concentrations (5, 25, 50, 100, 250, 500 μM, and 1.0 mM) of each peptide for 24 hours, and the cell viability was analysed using the lactate dehydrogenase (LDH) assay. There was no statistically significant difference between the viability of the control (untreated) cells and the cells exposed to the peptides. None of the peptides exhibited cytotoxic effects at the concentrations tested (data not shown). In addition, peptides at a 1.0 mM dosage did not exhibit any side effects on MDV-uninfected or infected embryos, including no effect on embryo activity and no apparent pathological changes.

### gHH1, gHH2, gHH3, gHH5, and gBH1 have potent antiviral effects on lesion formation

**Figure 4:**
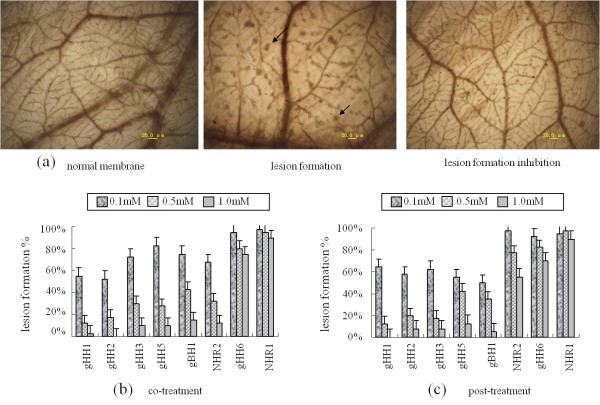
**Pathological reduction by treatment of MDV with peptides in chicken embryos**. **(a) **Photos show pathological changes induced by treatment of virus with or without peptides from chicken embryos (at 10 × 20 magnification, arrows indicate gray lesions). These representative images show normal membrane (no treatment), 100% lesion formation (infected by 10^3 ^pfu of MDV without peptides) and 0% lesion formation (10^3 ^pfu of MDV with 1.0 mM gHH2 in co-treatment assay, i.e., 100% lesion formation inhibition), respectively. **(b and c) **The infectivity rates (proportion of lesion formation) in the presence of different peptides at various concentrations (0.1, 0.5, 1.0 mM) for co-treatment (Fig. 4b) and post-treatment (Fig. 4c) assays. Peptides gHH1, gHH2, gHH3, gHH5, gBH1, NHR2, gHH6, and NHR1 are shown from left to right, respectively.

We next examined the antiviral effects of these peptides on chorioallantoic membrane (CAM) lesion formation. Briefly, various concentrations of peptides (0.1, 0.5, or 1.0 mM) in 60 μl of solution were mixed with 10^3 ^pfu of virus and injected into the yolk sacs of 6-7-day-old embryonated chicken eggs (for the co-treatment assay), or yolk sacs were infected with virus for 1.5 hours at 37°C before peptides were administered (for the post-treatment assay). After 9 days, the CAMs were harvested and lesion formation was measured. The mean of 63 no-peptide control lesions per membrane was determined to be very reproducible. All peptides showed dose-dependent activity against lesion formation, and gHH1 and gHH2 were particularly effective at inhibiting infection in both virus co-treatment and post-treatment assays. Lesion formation was completely inhibited by the gHH1 peptide in the post-treatment assay and by gHH2 in the co-treatment assay at a concentration of 1.0 mM. Interestingly, NDVHR2 at 1.0 mM showed effective antiviral activity against MDV only in the co-treatment assay with reduction of lesion formation to 15%. In addition, the non-specific protein NDVHR1 and gHH6 did not show significant antiviral activity in either assay. These results are presented in Figure 4.

### Structural characteristics of gHH1, gHH2, gHH3, gHH5, and gBH1 peptides

**Figure 5:**
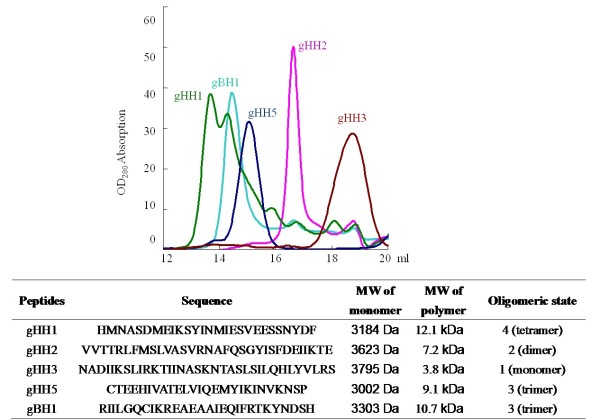
**GF results and structural analyses of five peptides**. **(top) **GF analysis of peptides. The purified peptides were loaded onto the Superdex G75 column in a buffer solution of 20 mM Tris-HCl, pH 8.0. The peak molecular mass was estimated by comparison with the protein standards running on the same column. The clear peak of gHH1 occurred at about 12.1 kDa. This molecular mass matches the approximate sum of four molecules, indicating the formation of the homotetrameric structure. The peak of gHH2 occurred at about 7.2 kDa, indicating the formation of the homodimeric structure. The peak of gHH3 occurred at about 3.8 kDa, indicating the monomeric state. The peak of gHH5 occurred at about 9.1 kDa, indicating the formation of the homotrimeric structure. The peak of gBH1 occurred at about 10.7 kDa, indicating the formation of the homotrimeric structure. **(bottom) **Sequences and calculated oligomeric states of gHH1, gHH2, gHH3, gHH5, and gBH1 in aqueous solution.

**Figure 6:**
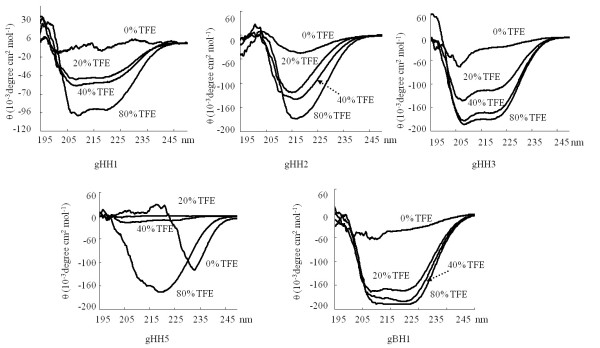
**CD spectroscopy analyses of peptides**. CD spectroscopic results of gHH1, gHH2, gHH3, gHH5 and gBH1 in PBS solution with 0%, 20%, 40%, 80% TEF, respectively. The gHH1, gHH3 and gBH1 peptides adopted a standard α-helical conformation with double minima at 208 nm and 222 nm in a PBS-buffered solution and remained practically unaltered in the presence of TFE. CD analysis of gHH2 showed that the peptide adopted a β-sheet conformation in buffer solution, and its CD spectra remained practically unaltered in TFE solution. gHH5 had a structural change from random coil to α-helical structure when the peptide was transferred from a polar environment to membrane interfaces using aqueous mixtures of TFE.

The structural characteristics of the five peptides that demonstrated protective effects in the cell and embryo infectivity assays were examined. Firstly, MS spectrometry showed that the molecular masses of gHH1, gHH2, gHH3, gHH5, and gBH1 were 3184, 3623, 3795, 3002 and 3303 Da, respectively. To investigate the structures of these peptides, gel filtration (GF) and circular dichroism (CD) spectroscopy analyses were performed. These results demonstrated that the majority of the peptides transitioned to the oligomeric state in the polar environments of Tris-HCl and lipidic solutions with 2,2,2 trifluoroethanol (TFE). Specifically, GF chromatography of gHH2 demonstrated the formation of a homodimeric structure with a molecular mass of about 7.2 kDa in aqueous solution. CD spectroscopy of gHH2 showed that the peptide adopts a β-sheet conformation in aqueous solution, and this tendency towards β-sheet formation becomes more obvious in a TFE solution. GF chromatography of gHH5 revealed a molecular mass of 9.1 kDa, suggesting the formation of a homotrimeric structure in polar environments. Analysis of gHH5 revealed a conformational change from a random coiled structure to a more obvious α-helical structure when the peptide was transferred from a polar environment to membrane interfaces using aqueous mixtures of TFE, suggesting the formation of potential higher-order oligomers in lipidic solutions. These results are presented in Figures 5 and 6.

For gHH1, gBH1, and gHH3, which share a similar α-helical secondary structure, GF chromatography and CD spectroscopy were performed under the same experimental conditions. GF chromatography of gHH1 demonstrates the formation of a homotetrameric structure with a molecular mass of about 12.1 kDa, matching the sum of four peptides. The gBH1 peptide forms a homotrimeric structure with a molecular mass of 10.7 kDa, which is approximately equal to the sum of three molecules; gHH3 adopts a monomer formation with a molecular mass of 3.8 kDa (see Figure 5). Results of CD analysis of gHH1, gHH3, and gBH1 show that all three peptides adopt a standard α-helical conformation with double minima at 208 nm and 222 nm in a PBS-buffered solution, and the tendency to form α-helices is clearer in the presence of TFE (see Figure 6).

## Discussion

In this study, eleven potential HR regions of MDV gH and gB were identified using GOR bio-software. These regions overlap with some α-helix-enriched regions, including gHH1, gHH3 and gBH1, and with hydrophobic regions, including gHH2 and gBH1 (data not shown). MDV glycoproteins have more HR regions than herpes simplex virus type 1 (HSV-1) and human cytomegalovirus (HCMV), which have only two HR regions in gH or gB [11,12]. Furthermore, five peptides (gHH1, gHH2, gHH3, gHH5, and gBH1) showed potent antiviral activity in a plaque formation assay using MDV-infected CEFs and were considered for further analysis (see Figure 2c). The plaque formation studies also demonstrated that the most active peptide, gHH1, was effective both against viral entry and after virus entry, while gHH2 was most effective in the virus pre-treatment assay (see Figure 2d). The inhibitory activity of the peptides may have occurred via the peptides associating with glycoproteins gH or gB to block the conformational changes of these glycoproteins that are crucial for fusion; it is also possible that these peptides may inhibit glycoprotein binding to receptors [13,23].

We used CEF-associated MDV instead of cell-free MDV in cell infectivity and embryo assays due to the need for consistent treatment in terms of virus titre at different times [24]. Ever since Woodruff and Goodpasture [25] first introduced the technique of cultivating fowlpox virus on the CAMs of a chicken embryo, this method has been widely used in studies of virus isolation (herpesvirus) and tissue invasion by viral transformed cells and been considered as a model system to screen drugs [26,27]. Due to the effect of serially passaged MDV on inducing varied lesions on CAMs, we used the same passage of CEF-associated MDV to study lesion formation and reduction after peptide treatment [28]. In addition, within a range of 50 to 80 lesions per membrane, a linear relationship exists between the number of lesions and the infecting virus dilution. Therefore, 10^3 ^pfu MDV that formed a mean number of 63 lesions was selected for use in this assay. In vivo assays may be performed by counting the number of lesions appearing on CAMs after a few days (i.e., 5-6 days) after their direct in vivo (DIO) inoculation [29] or 10-14 days after inoculation of yolk sacs of 9-10-day-old eggs [30,31]. In our study, infective inoculum was inoculated into yolk sacs of 6-7-day-old chicken embryos. After 9 days of additional incubation, surviving embryos were monitored for lesion formation. Although the egg still alive until day 10-13 after infection, the lesion size on CAMs was sometimes inconsistent (e.g., there was separation into large and small lesions). As a result, we used 9 days post-infection as a constant observation time, which gave the results considerable precision. The tested peptides showed potent antiviral activity in the embryo assay, and both gHH1 and gHH2 were very effective in co-treatment and post-treatment assays at a concentration of 1.0 mM (see Figure 4). Further experiments will examine the infection of maternal antibody-free 1-day old chickens (SPF chickens) with a pathogenic strain of MDV with or without peptides to study the role of these peptides on the pathogenesis of MDV.

Much established evidence has shown that the HR2 regions of fusion glycoproteins from enveloped viruses have potent and specific antiviral activities [1]. Our previous research demonstrated that the HR2 from NDV (i.e., avian paramyxovirus-1, APMV-1) is a specific inhibitor of NDV membrane fusion that has no cross-inhibitory activity against APMV-2 [32]. Some reports on the HR region of bovine herpesvirus type 1 (BoHV-1) have shown infection inhibition activity, which was obtained not only with other herpesviruses but also partly with NDV [33]. In this study, we tested a highly effective inhibitor of NDV infection for its ability to inhibit the infectivity of the unrelated MDV. The results of cell infectivity and embryo assays indicated that NDVHR2 exerted effects on MDV in the specific stage of virus entry (i.e., in cell pre-treatment and embryo co-treatment assays), suggesting a potential cross-inhibitory effect of NDV HR2 in MDV infection. In addition, NDVHR1 did not exert any effect on MDV infection, supporting the specificity of the antiviral effect of the MDV-derived peptides in this paper (see Figures 2 and 4).

We further studied the structures of the peptides used in this study. The three-dimensional (3-D) structure of HSV-2 gH shows three distinct domains: the N-terminal domain that binds gL (H1 domain), the central helical domain (H2 domain) and the C-terminal β-sandwich domain (H3 domain). Six MDV gH-derived peptides (gHH1, H2, H3, H4, H5, and H6) are within the H2 domain, which is globular and mostly helical. The H2 domain contains thirteen α-helices and three short 3_10 _helices. In addition to the helices, this domain has a β12 strand that participates in a six-stranded β-sheet within the H1B subdomain and a short β11 strand that makes a small antiparallel β-sheet with the β4 strand of the H1B subdomain [5]. In the current study, results of GF and CD analyses showed that MDV-gHH1 adopts a homotetrameric structure with a standard α-helical conformation, consistent with the 3-D result and this tendency to form α-helices is more obvious in the presence of TFE. The ratio of ellipticities at 222 and 208 nm can be utilized to distinguish between the monomeric and oligomeric states of helices [13]. When the θ222/θ208 ratio is approximately 0.8, the peptide is in its monomeric state, and when this ratio exceeds 1.0, the peptide is in its oligomeric state. The CD data from MDV-gHH1 reveals that gHH1 undergoes a conformational change from the oligomeric state to a monomer/oligomer equilibrium, following which it shifts towards the monomeric state with increasing concentrations of TFE (see Figures 5 and 6). Furthermore, amino-acid alignment analysis was employed to compare the corresponding domains of MDV-gHH1 with those in other α-herpesviruses. No significant antiviral activity was found in published reports. The MDV-gHH2 has a homodimeric structure and adopts a β-sheet conformation in aqueous solution, and this β-sheet tendency is more obvious in TFE solution, as highlighted by the fact that MDV-gHH2 has a more obvious tendency to oligomerize in membrane interfaces (see Figures 5 and 6). MDV-gHH2 may be important as a binding site for glycoprotein receptors, given its potent antiviral activity, its performance in the virus pre-treatment assay, and its high propensity for interfacial hydrophobicity. The secondary structure of gHH2 is similar to that of the HSV-1 internal fusion peptide (IFP) region (a.a. 377 to 397), from which the ability to partition into membranes and aggregate within them arises [16]. However, the domains of HSV-1 a.a. 381-420 which correspond to MDV-gHH2 did not show any significant antiviral activity [23]. Two HSV-1 peptides, a.a. 493 to 512 and a.a. 626 to 644 of HSV-1 gH, are homologous to MDV-gHH4 and MDV-gHH6, respectively. Both peptides showed highly antiviral activity and exhibited membranotropic characteristics [23,34]. However, MDV-gHH4 and MDV-gHH6 did not show potent antiviral activity in our study. It is worth noting that the gHH1, H2, H4, and H6 peptides of MDV gH showed different antiviral functions from the corresponding domains derived from HSV-1 gH. In fact, only 23% residue identities exist between MDV gH and HSV-2 gH, and no existing analytical tools can predict the structure of MDV gH according to the resolved 3-D structure of HSV-2 gH.

It has been established that both the HR1 (a.a. 444 to 479) and HR2 (a.a. 542 to 582) domains of HSV-1 gH show potent antiviral activity in cell infectivity assays [11]. These domains were recently studied by Chowdary et al. using x-ray crystallography. These authors' results indicated that the pre-fusion structure of HSV-2 gH did not reveal any domains with heptad repeat (HR) characteristics [5]. The trimeric hairpin bundle, which was suggested to be characteristic of the post-fusion form of class I and class III fusogens, is absent from the gH structure, although these two domains can form helical bundles. Given that gH appears to be able to mediate cell-cell fusion in some herpesviruses, we cannot exclude the possibility that gH has some intrinsic fusogenic properties [10-16,34]. It is possible that the conformation of gH could change during the fusion process or viral entry to expose heptad repeats not observed in the pre-fusion structure. The results of CD analysis of MDV-gHH3 (homologous to HR1 of HSV-1 gH) in the present study showed that the peptide adopts a standard α-helical conformation and that there was no effect of polarity on the monomeric state when the peptide was transferred from polar to non-polar membrane environments, similar to the GF result in aqueous solution. MDV-gHH5 (homologous to HR2 of HSV-1 gH) revealed the formation of a homotrimeric structure in polar environments and the formation of α-helical structure in lipidic solutions (see Figures 5 and 6). More importantly, our study revealed that both MDV-gHH3 and MDV-gHH5 show potent antiviral activity, not only in plaque formation assays (in vitro) but also in embryo assays (in vivo) (see Figures 2 and 4), further supporting the idea that these peptides have fusogenic properties involved in the viral entry process.

**Figure 7:**
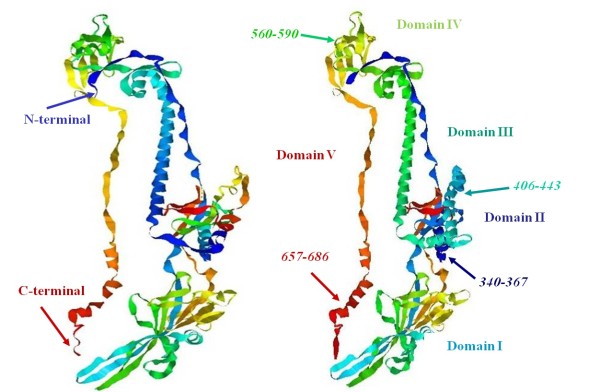
**Schematic representation of the locations of the peptides on the structures of HSV-1 and MDV gB**. **(left) **Three-dimensional structure of HSV-1 gB ectodomain. The structural coordinates were obtained from http://www.pdb.org/pdb/home/home.do. The structure is designated as 2GUM in the PDB and was visualized using the visual molecular dynamics program at http://www.ks.uiuc.edu/Research/vmd. **(right) **Predicted three-dimensional structure of the MDV gB ectodomain. The homology model of MDV gB was predicted by modelling against the known structure for HSV-1 gB using Swiss-Model via the ExPASy Web server http://swissmodel.expasy.org. Five domains observed in the crystal structure are highlighted in different colours and the corresponding positions of gBH1, H2, H3, and H4 are shown.

Based on the gB crystal structure, the gB trimer can be divided into six distinct structural domains, and four functional regions (FR) have been defined based on the mapping of anti-gB neutralizing MAbs to the crystal structure [4]. In the current study, the MDV-gBH1 in domain II adopted an α-helical conformation in 20% TFE solution with the monomer/oligomer equilibrium shifting toward the oligomeric state in 40% TFE. At higher concentration of TFE of 80%, the ratio of ellipticities at 222 and 208 nm decreases to approximately 1.0, indicating a monomer/oligomer equilibrium state (see Figures 5 and 6). HSV-1 gB389-398, which is homologous to gBH1, is unable to induce lipid mixing in this assay condition and did not significantly inhibit infection in another study [15]. However, MDV-gBH1 showed higher antiviral activity, not only in vitro but also in vivo. As for other gB-derived peptides, a recent study by Atanasiu et al. suggested that FR2 in domain II (the main epitopes of FR2 lies within residues 454 to 473 homologous to gBH2) plays a critical role in the interaction between gB and gH, and the gB binding site is considered to be an attractive target for antiviral design [35]. In our study, MDV-gBH2 did not show significant antiviral activity. This result suggests that residues involved in the interaction between gB and gH are not essential for membrane fusion. In fact, the predicted MDV-gBH2 structure is quite different from that of HSV-1 gB (see Figure 7). It is worth noting that HR1 (a.a. 477 to 510) and HR2 (a.a. 696 to 724) from gB of bovine herpesvirus type 1 (BoHV-1) have been studied [33], and only the former consistently inhibited virus replication. In the current study, a.a. 467 to 500 (homologous to HR1 of BoHV-1gB, data not shown) in domain III, MDV-gBH3 in domain IV and MDV-gBH4 (homologous to HR2 of BoHV-1gB) in domain V did not show meaningful antiviral activity. MDV gB-derived peptides clearly showed different antiviral functions from the corresponding domains derived from gB peptides of other α-herpesviruses. Figure 7 (right) schematically presents the locations of the potential inhibitory peptides on the MDV gB ectodomain.

Membrane fusion is an important step in enveloped virus entry into host cells. The present study on the antiviral activity of MDV-derived peptides that are involved in the viral entry process reveals viral entry mechanisms. These peptides may be also useful as small-molecule antiviral inhibitors. It is notable that some peptides were able to block viral infection at a post-attachment entry step, suggesting that the peptides would likely be useful as oral preventive agents or as microbicides. Further studies are needed to better define the precise mechanisms of inhibition of these peptides and the specific nature and location of their interactions with viral targets. Additional issues concerning the similarities and differences between the membrane fusion mechanisms of MDV and other α-herpesviruses should also be addressed.

## Methods

### Prediction and analysis of fusogenic regions

The combined use of biological software prompted us to analyse the different domains of gH (GenBank Accession No. AAL37975) and gB (GenBank Accession No. AAM97699) of MDV strain RB1B in detail for potential membrane fusion-related regions. Biological software package ExPASy-Coils http://www.ch.embnet.org/software/coils/COILS_doc.html, which has been successfully used to analyse a number of viral fusion proteins, was used to study coiled-coils (see Figure 1). We chose the ExPASy-tools program (GOR software, http://www.ch.embnet.org/, as it was designed specifically to analyse secondary structures. Hydropathy plots corresponding to the sequences of MDV gH and gB were obtained using TMpred (ExPASy, Swiss Institute of Bioinformatics, http://www.ch.embnet.org and Membrane Protein eXplorer (MpeX, Stephen White laboratory, http://blanco.biomol.uci.edu/mpex). In particular, hydropathy plots were obtained using the hydropathy index of Kyte and Doolittle and the interfacial hydrophobicity scales of Wimley and White for individual residues.

The peptides from MDV gH, identified as H1, H2, H3, H4, H5, and H6, are located at amino acid (a.a.) residues 277 to 303, 331 to 362, 396 to 429, 434 to 467, 508 to 533, and 576 to 604, respectively. The peptides from MDV gB, identified as H1, H2, H3, H4, and H5, are located at a.a. residues 340 to 367, 406 to 443, 560 to 590, 657 to 686, and 775 to 883, respectively.

### Primer design and gene construction

All genes were constructed using the bridging PCR method and cloned into the GST fusion expression vector pGEX-6P-I at the *BamH*I-*Xho*I restriction sites where there is a rhinovirus 3C protease cleavage site for the fusion protein (as in the commercial PreScission™ protease cleavage site). The positive plasmids were verified by direct DNA sequencing.

### Protein expression and purification

*Escherichia coli *strain Ros, transformed with the recombinant pGEX-6p-I plasmid, was grown at 37°C in 2 × YTA to an optical density of 0.8-1.0 (OD at 590 nm) before being induced with 1 mM IPTG for 4 hours. Bacterial cells were harvested and lysed by sonication in PBS (pH 7.3). Triton X-100 was then added to a final concentration of 1% and the lysate was incubated for 30 min at 0°C. The clarified supernatants were passed through a Glutathione-Sepharose 4B column. The GST fusion protein-bound column was washed with over 10 column volumes of PBS and eluted with 3 column volumes of reduced glutathione. The GST fusion proteins were then cleaved by GST fusion rhinovirus 3C protease at 5°C for 16 hours in a 50 mM Tris-HCl buffer, pH 7.0. The cleaved proteins were then purified by affinity filtration (with the Glutathione-Sepharose 4B column) following which the column-unbound protein was extracted and concentrated by ultrafiltration with 3K membranes (Millipore). The resultant protein was dialyzed against PBS, reduced to a proper concentration by ultrafiltration and stored at -70°C for further analysis. GST fusion proteins and cleaved proteins were analysed by 15% tricine SDS-PAGE.

### Preparation of MDV stock

Primary chicken embryo fibroblasts (CEFs) were grown in DMEM supplemented with 10% foetal calf serum (FCS) and were allowed to attach overnight. CEF-associated MDV strain RB1B (from Shandong Agriculture University, has been passaged multiple times in primary CEFs) was incubated for 2 hours at 37°C. Following incubation, the virus samples on the cells were replaced with DMEM supplemented with 2% FCS and the cultures were incubated for another 5 days [24,36]. Consistent and uniform plaques were observed and counted under an Olympus microscope and images were captured using DP Controller software. CEF-associated MDV from the same passage at 2 × 10^4 ^plaque forming units (pfu) was used in both cell infectivity and chicken egg assays in this study.

### Effect of the peptides on plaque formation

All of the peptides were dissolved in DMEM without FCS and used at a range of concentrations. For the antiviral activities of peptides in the co-treatment assay, 100 pfu of MDV was incubated with the peptide at different concentrations for 2 hours at 37°C. A no-peptide sample control was also prepared and this sample was regarded as 100% plaque formation. Following incubation, the virus-peptide mixtures on the cells were replaced with DMEM supplemented with 2% FCS and the cultures were incubated for 5 days. At the end of this incubation, 50% inhibitory concentrations (IC_50_) values were calculated.

To assess the effects of peptides with IC_50 _values below 12 μM on the inhibition of MDV infectivity, four different methods [13,23] of treating cell monolayers were used: 1) Virus pre-treatment − virus was incubated in the presence of peptides at 25 μM for 1 hour at 37°C and was then titrated onto cell monolayers; 2) Cell pre-treatment − cells were incubated with peptides for 30 minutes at 4°C. Peptides were removed, and cells were washed with PBS. Following this treatment, the cells were infected with MDV; 3) Co-treatment − the cells were incubated peptides in the presence of viral inoculum for 1 hour at 37°C; and 4) Post-treatment − cell monolayers were infected with virus for 45 minute at 37°C. The peptides were then added to the inoculum, followed by an additional 30 minute incubation at 37°C. Monolayers were incubated for 5 days at 37°C in DMEM supplemented with 2% FCS. The ratio of plaque counts to the no-peptide sample control is reported as the percentage of plaque formation (by arithmetic conversion of the mean percent plaque formation). Results are expressed as the average of triplicates ± the standard deviation and all experiments were conducted in parallel with each peptides and non-specific peptides.

### LDH assay for toxicity analysis

Peptide cytotoxicity was measured using the lactate dehydrogenase (LDH) assay. This assay was performed according to the manufacturer's instructions using a commercial cytotoxicity detection kit (Roche).

### Virus-yield reduction assay in chicken embryos

Briefly, 1 × 10^3 ^pfu of CEF-associated MDV, was injected into yolk sacs of 6-7-day-old embryonating specific-pathogen free (SPF) chicken eggs. After 9 days of additional incubation, surviving embryos were chilled overnight at 4°C and observed for lesion formation.

In the co-treatment protocol, a mixture of the MDV inoculum (1 × 10^3 ^pfu) with various concentrations of peptide (0.1, 0.5, 1.0 mM) in 60 μl of solution was injected into the yolk sacs of chicken eggs and incubated at 37°C for 9 days. For post-treatment assays, the yolk sacs were infected with virus for 1.5 hours at 37°C and then peptides were administered over a range of concentrations for 9 days. The chorioallantoic membranes (CAMs) at day 9 post-incubation were fixed in 10% buffered formalin. Lesions (pox) were observed and counted under an Olympus microscope, and lesion images were captured using DP Controller software. The ratio of lesion counts to the no-peptide sample control is presented as the percentage of infection (by arithmetic conversion of the mean percent lesion formation). Results are expressed as the average of triplicates ± the standard deviation and all experiments were conducted in parallel with each peptides and non-specific peptides. Five embryos were used in each experiment to generate small standard errors in the assay.

### Mass spectrometry (MS) analyses

All of the purified cleaved peptides were resolved in a 20 mM Tris-HCl, pH 8.0 buffer and then analysed using the Bruker Daltonics *Biflex *III MALDI-TOF Mass Spectrometer to ascertain the molecular masses of the peptides.

### Gel filtration (GF) analyses

The purified cleaved peptides were loaded onto the Superdex G75 column in a solution buffer of 20 mM Tris-HCl, pH 8.0. The peak molecular mass was estimated by comparison with protein standards running on the same column. The peak fractions were collected and analysed by 15% SDS-PAGE. The analytical column was calibrated using a series of individual runs of standard molecular mass proteins as markers including bovine serum albumin (68 kDa), egg white albumin (43 kDa), ribose nucleotidase (13.7 kDa), aprotinin (6.5 kDa), antimicrobial peptides (5 kDa), and vitamin B12 (1.4 kDa).

### Circular dichroism (CD) spectroscopy analyses

The purified, cleaved peptides were dissolved in 10 μM PBS, pH 7.4 with 20%, 40%, or 80% 2,2,2 trifluoroethanol (TFE). The wavelength-dependence of molar ellipticity [θ] was monitored at 25°C as the average of eight scans in a spectropolarimeter (Model J-710) equipped with a thermoelectric temperature controller. The TFE solution was obtained from Fluka (Sigma-Aldrich, Milan, Italy) and was prepared using distilled water. The buffers were also filtered in a vacuum pump system using 0.2 μm pore membrane filters. TFE is widely used in conformational studies because it promotes intramolecular hydrogen bonds in spite of intermolecular interactions with water molecules. Moreover, as TFE lowers the polarity of the solution, the environmental changes explored by the peptides resemble those of the native sequences during the membrane fusion process [13].

## Competing interests

The authors declare that they have no competing interests.

## Authors' contributions

XW conceived of the study, performed a design of experiments and the bio-software analysis. XC carried out the structural characteristics studies, participated in plaque and lesion formation assays. MW participated in statistical analysis and coordination. All authors read and approved the final manuscript.
